# Acetylcholine regulation of GnRH neuronal activity: A circuit in the medial septum

**DOI:** 10.3389/fendo.2023.1147554

**Published:** 2023-03-06

**Authors:** David M. Shostak, Stephanie Constantin, Jill Flannery, Susan Wray

**Affiliations:** Cellular and Developmental Neurobiology Section, National Institute of Neurological Disorders and Stroke/National Institutes of Health, Bethesda, MD, United States

**Keywords:** acetylcholine, nicotinic, muscarinic, cholinergic receptors, calcium imaging, GnRH neurons, medial septum (MS)

## Abstract

In vertebrates, gonadotropin-releasing hormone (GnRH)-secreting neurons control fertility by regulating gonadotrophs in the anterior pituitary. While it is known that acetylcholine (ACh) influences GnRH secretion, whether the effect is direct or indirect, and the specific ACh receptor (AChR) subtype(s) involved remain unclear. Here, we determined 1) whether ACh can modulate GnRH cellular activity and 2) a source of ACh afferents contacting GnRH neurons. Calcium imaging was used to assay GnRH neuronal activity. With GABAergic and glutamatergic transmission blocked, subtype-specific AChR agonists and antagonists were applied to identify direct regulation of GnRH neurons. ACh and nicotine caused a rise in calcium that declined gradually back to baseline after 5-6 min. This response was mimicked by an alpha3-specific agonist. In contrast, muscarine inhibited GnRH calcium oscillations, and blocking M2 and M4 together prevented this inhibition. Labeling for choline acetyltransferase (ChAT) and GnRH revealed ChAT fibers contacting GnRH neurons, primarily in the medial septum (MS), and in greater number in females than males. ChAT positive cells in the MS are known to express p75NGFRs. Labeling for p75NGFR, ChAT and GnRH indicated that ChAT fibers contacting GnRH cells originate from cholinergic cells within these same rostral areas. Together, these results indicate that cholinergic cells in septal areas can directly regulate GnRH neurons.

## Introduction

Acetylcholine (ACh) modulates reproductive function, acting both peripherally (1) and centrally (2). Centrally, measurements of ACh in the hypothalamus showed sexual dimorphism as well as changes during the estrous cycle (3–5). In the preoptic area of the hypothalamus (POA) in male rats, cholinergic axons contact gonadotropin releasing hormone (GnRH) neurons (6), which control release of gonadotropins from the anterior pituitary and subsequently gonadal function. To date, the direct impact of ACh on GnRH neuronal activity, the ACh receptor (AChR) subtype(s) expressed by GnRH neurons, as well as the source of the cholinergic afferents to GnRH neurons are unclear.

AChRs are divided into two groups: metabotropic acetylcholine receptors activated by muscarine (mAChRs), and ionotropic acetylcholine receptors activated by nicotine (nAChRs) (7, 8). *In vivo* and *in vitro* studies examined the role of ACh in the regulation of GnRH/luteinizing hormone (LH) secretion. ACh application into the POA (containing GnRH neurons) evokes LH secretion, and the effect is partially reduced by atropine, a non-selective mAChR antagonist (9).

Similarly, intracerebroventricular atropine blocks the preovulatory LH surge (10) while POA electrostimulation, which triggers GnRH secretion, restores it (11). This indicates the central activation of mAChRs is required for GnRH/LH secretion and ovulation. Yet, atropine infusion directly in the POA does not block the preovulatory LH surge (12) but pilocarpine (non-selective mAChR agonist) infusion in the POA does (13). Although the precise mechanisms were not identified, these studies indicate that central mAChRs are involved in the GnRH/LH surge and subsequently ovulation. Work from rat hypothalamic cultures (14) and the mouse-derived GT1-7 GnRH cell line (15–17) showed that the nicotinic system can modulate the release of GnRH. Both ACh and nicotine stimulate GnRH release while muscarine inhibits it. In the GT1-7 cell line, alpha7 nAChR drives the stimulation (15, 17) while M2 mAChR drives the inhibition (16). However, transcripts for other nAChR subunits and mAChR receptors were also detected (15). Thus *in vivo* and *in vitro* data suggest that ACh can modulate GnRH secretion, possibly by direct action of ACh on GnRH neurons, *via* nAChRs and mAChRs. Yet, the regulation of primary GnRH neurons by ACh remains unknown (18).

In this study, we use primary GnRH neurons maintained in explants, devoid of ACh neurons, to uncover the mechanism(s) underlying cholinergic regulation of GnRH neuronal activity and examine GnRH neurons *in vivo* for the presence and source of cholinergic afferents. Using calcium imaging, we show that ACh causes a transient rise in intracellular calcium levels, *via* alpha3-nAChR and subsequent opening of voltage gated calcium channels (VGCC). In contrast, ACh decreases the frequency of calcium oscillations, *via* M2/M4 mAChRs and subsequent opening of G-protein coupled inward-rectifying potassium (GIRK) channels. Finally, we show that cholinergic fibers contact GnRH neurons *in vivo*, primarily in the medial septum (MS), in a sex- and age- dependent manner.

## Methods

### Animals

All procedures were approved by National Institute of Neurological Disorders and Stroke, Animal Care and Use Committee and performed in accordance with National Institutes of Health guidelines. PN10, PN35 and Adult NIH Swiss, GnRH-GFP (MGI:6158458)/ChAT-Cre (MGI:5475195)/Rosa26^tdTomato^ (MGI:3809523) were used. At least 3 female and male mice were used at all ages. The stage of the estrous cycle was not determined in adult females. PN10 mice were euthanized using CO_2_ asphyxiation followed by cervical dislocation. The head was quickly decapitated, skin removed, and two cuts were made to allow optimal fixation of brain tissue; one from caudal to rostral along the top of the skull, and another coronally to remove the nose. PN10 brains were stored in 4% formaldehyde in PBS for two nights, then placed in a 30% sucrose-PBS solution for two nights. PN35 and Adult mice were anesthetized with isoflurane then killed using an intraperitoneal overdose of ketamine (20 mg/20 g). Mice were transcardially perfused with 0.1 M PBS followed by 4% formaldehyde in PBS. The brain was removed, placed in 4% formaldehyde in PBS for an additional hour, then transferred to a 30% sucrose-PBS solution for two nights. All tissue was frozen using dry ice and kept at −80°C. Coronal sections (40 µm) were cut with a sliding freezing microtome from the olfactory bulbs to the median eminence and stored at −20°C in cryoprotectant (19).

### GnRH neurons in explants

Primary GnRH neurons maintained in nasal explants were cultured as previously described (20). Both genders were included in all experiments since the sex of the embryos used to make explants was not determined. One embryo gives one explant. For any given treatment, explants generated from at least 2 different litters were used as well as from two different generation dates. Nasal explants are a useful model to unravel the mechanisms underlying GnRH physiology. An important advantage of this model is that whole GnRH neurons are present, both cell body and processes. In addition, many endogenous brain factors are absent, allowing direct assessment of the perturbation on GnRH neuronal activity. Similar to GnRH cells examined in brain slices of adult animals, bursts of action potentials occur concomitantly with [Ca2+]i oscillations in GnRH neurons maintained in explants (21). Notably, GnRH cells maintained in explants express many of the receptors displayed by GnRH cells in brain slices from adults [see (22)] and Ca2+ imaging has been successfully used to assess both GnRH neuron function and coordination, as numerous GnRH cells (30-50 cells) can be imaged simultaneously (21). Briefly, 11.5-day old embryos were acquired from time-mated NIH Swiss females. Nasal pits were dissected in Gey’s Balanced Salt Solution (Life Technologies. Carlsbad, CA) supplemented with 5mg/mL glucose (Sigma-Aldrich, St. Louis, MO) then adhered to Permanox coverslips (Nunc, Rochester, NY) using a plasma (Cocalico Biologicals Inc, Reamstown, PA)/thrombin (Sigma-Aldrich) clot. Explants, cultured in a humidified incubator (37°C, 5% CO_2_) using a serum-free medium (SFM), were treated with fresh SFM containing 2.3 μM fluorodeoxyuridine (Sigma-Aldrich) between days 3 and 6 to inhibit the proliferation of non-neuronal tissue. On day 6, and every 2 days afterward, the medium was replaced with fresh SFM. Explants were used for experiments between 8-11 days *in vitro* (div).

### Single GnRH cell PCR verification

Three’ biased Poly(A)-amplified cDNA library from individual GnRH neurons from non-treated explants were used for analysis of transcripts in single cells (23). Each single-cell cDNA was tested for GnRH transcript with PCR. Primers were designed for the CHRNA3 (alpha3 nAChR), CHRM2 (M2 mAChR), and CHRM4 (M4 mAChR) genes within 500 bp of the polyadenylation site and verified for specificity using NCBI BLAST (Basic Local Alignment Search Tool) (24). Mouse brain cDNA and water were used as positive and negative controls, respectively. PCR for GnRH, alpha3 nAChR, and M2 mAChR reactions consisted of adding 10x Goldtaq PCR buffer, 1.5-2 mM MgCl2, 100-250 µM each deoxynucleotide mix (Life Technologies), 400 nM forward primer, 400 nM reverse primer, and 2.5 U AmpliTaq Gold (Life Technologies) to 1 µL template cDNA. The protocol for GnRH, nAChR, and M2 mAChR was performed as follows: initial 10 min denaturation (94°C), followed by 40, 40, or 45 cycles respectively with denaturation 30 s (94°C), annealing for 30 s (55, 57.9 or 60°C), and extension for 2 min (72°C), followed by 10 min post-elongation at 72°C. PCR for the M4 mAChR utilized Platinum Taq, consisting of 5x Platinum Superfi Mix, 200 µM each deoxynucleotide mix (Life Technologies), 500 nM forward primer, 500 nM reverse primer, 0.6 U GC Enhancer (Invitrogen), and 2.5 U Platinum Superfi DNA Taq Gold (Life Technologies) added to 1 μL template cDNA. The protocol was performed as follows: an initial 30 s denaturation (94°C), 45 cycles with denaturation 15 s (94°C), annealing for 15 s (62.2°C) and extension for 15 s (72°C), followed by 10 min post-elongation at 72°C. All PCR products were run on a 2% agarose gel. The correct band size was seen in all products along with the brain cDNA control, with no bands present in the water control. Primer sequences and annealing temperatures are listed in **Table 1**.

**Table 1:** Primer Sequences.

Gene (NCBI Ref.)	Primer Sequences (5’-3’) Forward/Reverse	Annealing Temperature (°C)	Product Size (base pairs)
alpha3 nAChR (Chrna3) (NM_145129.3)	F: ACCCAGAATGCAGGACGTTG R: CTCTGTGGACTCTAGGAGTTGTGG	60	114
M2 mAChR (Chrm2) (NM_203491.3)	F: TGTGGTCAGAGTGTGTCTTGG R: AAAACCCCTTAATTGCACGTT	58	106
M4 mAChR (Chrm4) (NM_007699.2)	F: GCCTGGACCAGAAACTCTTG R: GATGGGGAAAGATGGACTGA	62.2	204
GnRH1 (NM_008145.3)	F: ACTGGTCCTATGGGTTGCGCCCTG R: GCCTGGCTTCCTCTTCAATCAGAC	55	180

### Calcium imaging

**Figure 1:**
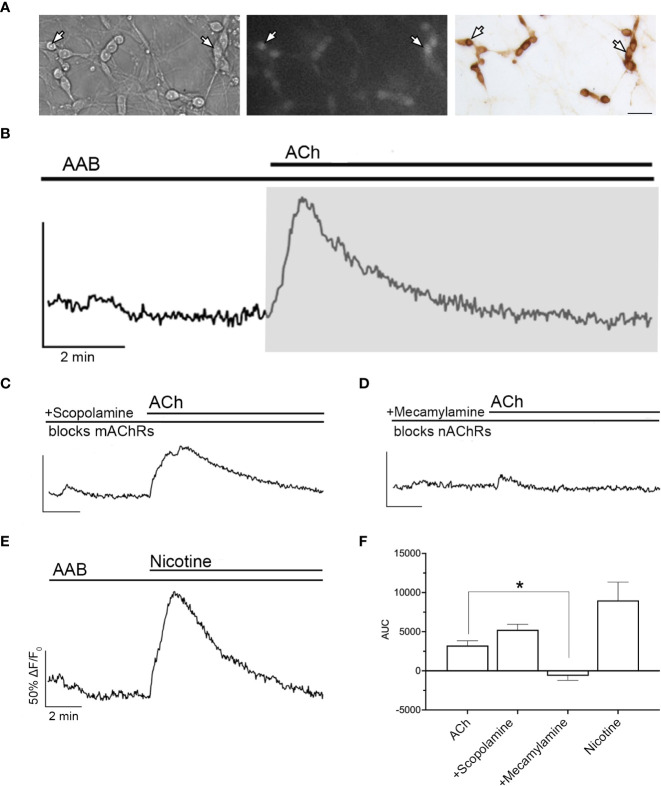
ACh-induced increase in [Ca^2+]^i occurs through nAChR. **(A)** Bright field image (left) was used to identify GnRH neurons (arrows). Cells maintained 8–11 days *in vitro* were loaded with calcium-sensitive dye Calcium Green-1 AM (middle) and imaged. Identity of cells as GnRH neurons was verified *post hoc* using immunocytochemistry (right). Scale bar, 25 µm. **(B)** Averaged trace from cells in single explant showing a transient increase in intracellular calcium levels ([Ca^2+^]i) with acetylcholine (ACh, 100 µM) in the presence in the presence of amino acid blockers (AAB), (BIC, 20 µM; CNQX, 10 µM; d-AP5, 20 µM), (n=155, N=6). Calcium responses were quantified by calculating the area under the curve (AUC, grey area) for the stimulation of nAChRs. Y-axis: the optical density (OD) expressed as ΔF/F_0_ (%), was normalized to 50% OD baseline over the 3 minutes prior to agonist application; X-axis: 2 min. **(C)** Blocking muscarinic receptors (mAChRs) with scopolamine (10 nM) did not prevent ACh-induced increase in [Ca^2+^]i (n=169, N=5). **(D)** Blocking nAChRs with mecamylamine (10 µM) did prevent ACh-induced calcium response (n=73, N=5). **(E)** Application of nicotine (100 µM) mimicked ACh-induced increase in [Ca^2+^]i, indicating that the calcium increase relies upon nAChRs activation (n=132, N=5). **(F)** Summary data showing the average AUC for **(B–E)**. Asterisks represent statistical significance between ACh (control) and other compounds (*p*<0.05, one-way ANOVA test with Dunnett’s multiple comparisons). All recordings display the mean of all explants **(N)**. Average number of GnRH cells analyzed/explant ranged from 15-34. (n = number of cells, N = number of explants).

Calcium imaging experiments were performed as previously described (21). Briefly, Calcium Green-1 AM (Life Technologies) was dissolved at 2.7 mM in dimethylsulfoxide (DMSO) containing 20% pluronic F-127 (Life Technologies), diluted to 13.5 µM in SFM (loading solution), aliquoted, and kept frozen until the day of the experiment. Explants were incubated in the loading solution for 20 min (37°C), then washed in fresh SFM for 20 min (37°C). Explants were then placed in a perfusion chamber and continuously perfused at ~300 μL/min with SFM at room temperature (~25°C) using a low-rate peristaltic pump (Instech Labs Inc, Plymouth Meeting, PA) coupled to a perfusion system (Warner Instruments LLC, Hamden, CT) allowing ‘in-line’ drug treatments. All experiments started and ended with SFM to confirm cell viability. Drugs were added sequentially, when possible, to evaluate their individual and/or additive effect. Explants were visualized (**Figure 1A** left, middle panel) using an inverted microscope (Eclipse TE2000-E, Nikon, Tokyo, Japan), through a 20X fluorescence objective [Fluor 20X; numerical aperture (NA), 0.75; working distance (WD), 1.0 mm] and a charge-coupled device camera (Retiga QImaging, Surrey, Canada) connected to a computer. Time-lapse recording was piloted by iVision imaging software (Scanalytics Inc, Fairfax, VA), and pictures were acquired at 0.5 Hz. Excitation wavelengths were provided by a Lumencor LED light engine (Beaverton, OR) going through a medium-width excitation bandpass filter at 465–495 nm, and emission was monitored through a 40 nm bandpass centered on 535 nm. The recorded field was chosen based on the bipolar morphology of the cells surrounding the tip of the nasal midline cartilage. The phenotype of the recorded cells was verified *post hoc* using immunocytochemistry for GnRH (see below, **Figure 1A**, right panel).

### Drugs

(−)-Bicuculline (BIC) chloride (20 µM, an A-type γ-aminobutyric acid [GABA] receptor antagonist), D-(−)-2-amino-5-phosphonopentanoic acid (AP5, 10 µM, a N-methyl-D-aspartate receptor antagonist), 6-cyano-7-nitroquinoxaline-2,3-dione (CNQX, 10 µM, AMPA/kainate receptor antagonist), tetrodotoxin citrate (TTX, 0.5 µM, a voltage-gated sodium channel blocker), acetylcholine (ACh, 100 µM, non-selective AChR agonist), scopolamine hydrobromide (10 nM, non-selective mAChR antagonist), mecamylamine hydrochloride (10 nM, non-selective nAChR antagonist), (−)-nicotine tartrate (100 µM, nAChR agonist), PNU282987 (10 µM, alpha7 nAChR agonist), TC2559 difumarate (10 µM, alpha4beta2 nAChR agonist), and CC4 (10 uM, alpha6beta2 nAChR agonist) were all purchased from Cayman Chemical (Ann Arbor, MI). Nifedipine (1 µM, L-type voltage-gated calcium channel blocker), (+)-muscarine chloride (Mus,10 µM, non-selective mAChR agonist), cesium chloride (Cs, 5 mM, broad-spectrum blocker of inwardly rectifying potassium (Kir) channels), methoctramine hydrate (50 nM, M2 mAChR antagonist) and phorbol 12-myristate 13-acetate (PMA, 50 nM, protein kinase C (PKC) activator) were purchased from Sigma-Aldrich. NS3861 (10 µM, alpha3beta2 and alpha3beta4 nAChR agonist) and PD102807 (400 nM, M4 mAChR antagonist) were purchased from Tocris (Bristol, United Kingdom).

### Analysis of calcium imaging

#### Nicotine

Experiments testing the effect of nicotine on GnRH neuronal activity consisted of a 25 min recording: SFM (5 min), amino acid blockers (AAB) ± TTX (5 min), AAB ± TTX+Agonist (10 min), SFM (5 min). The optical density (OD) of each recorded cell was determined over time, normalized to the mean OD (defined as 50%) of the last 3 last minutes of AAB or TTX treatment prior to application of agonist. Individual cell values from each explant were averaged and the area under the curve (AUC) for each explant was determined during the 10-min agonist period. A nAChR-evoked response for a given paradigm was defined by a *p* value < 0.05 using a one sample *t*-test with hypothetical value of 0. A Student’s *t*-test were used to compare AUC from two datasets. An one-way ANOVA followed by Dunnett’s multiple comparison test was used to compare AUC from multiple datasets to a given control. Statistical significance was set at p<0.05 in both cases.

#### Muscarine

The experiments testing the effect of muscarine on GnRH neuronal activity consisted of a 15 min recording to identify cells responding to muscarine, followed by a 20 min period to determine the receptors underlying the muscarinic response: SFM (5 min), AAB (5 min), Muscarine (5 min), SFM (5 minutes), AAB+Antagonist (5 min), AAB+Antagonist+Muscarine (5 min), SFM (5 min) and KCl (1 min) to verify cell viability. To determine if a cell was inhibited by muscarine, spontaneous variations in the frequency of calcium oscillations (peaks/min) were examined in 249 cells from 11 explants maintained in AAB and the standard deviation of 0.53 was observed. A decrease in the frequency of calcium oscillations ≥0.6 peaks/min was chosen as the threshold between spontaneous variations in the frequency of calcium oscillations (which accounted for ~85% (210 of 249) cells during the AAB control) and specific inhibition. Based on this, cells that decreased activity by ≥0.6 peaks/min in response to the first muscarine application were chosen for further analysis during the Antagonist+Muscarine period. The frequency of calcium oscillations from the selected cells were averaged for each period. A mAChR-evoked response for a given antagonist was defined by a *p* value <0.05 using a Student’s paired *t*-test between the 2 consecutive treatment periods of interest.

#### Verification that cells selected for calcium imagining were GnRH cells

Once calcium imaging was completed, explants were fixed in 4% formaldehyde (0.1 M PBS, pH 7.4, 30 min), washed in PBS, then kept at 4°C in cryoprotectant until processing. For staining, explants were washed in PBS, placed in blocking solution (10% normal horse serum/0.3% Triton X-100/0.1% NaAzide) for 1 h, washed in PBS, then incubated overnight at 4°C in an anti-GnRH primary rabbit antibody (in PBS with 1% BSA/0.1% NaAzide; SW-1, RRID:AB_2629221). The following day, explants were washed in PBS, incubated in a biotinylated secondary donkey anti-rabbit antibody (1:500 in PBS/0.3% Triton X-100; Jackson Immunoresearch) for 2 h, washed again in PBS, then processed for avidin–biotin horseradish peroxidase/3,3´-diaminobenzidine using standard procedures (DAB, brown, **Figure 1A**, right panel).

### Co-expression of M2 AChRs on GnRH cells

Explants (8–11 d) were fixed, washed, and blocked as described above, then incubated (2 nights, 4°C) in mouse monoclonal anti-M2 (10 µg/ml, DSHB: RRID:AB_2753215) mouse primary antibody. The brains of WT adult mice were perfused and cut as described above. Sections were washed in PBS (1 h), placed in a blocking solution (as above) for 2 h, washed again in PBS (1 h), then placed in FAB fragment anti-mouse IgG (1 h, Jackson Immunoresearch) to reduce non-specific binding of the secondary. Sections were washed again (30 min), fixed (20 min, 4% formaldehyde), washed (30 min), then incubated at 4°C for three nights in mouse monoclonal anti-M2 (2 µg/ml, DSHB: AB_2753215). Control explants or sections were incubated in bovine serum albumin (10%BSA). Explants/Sections were washed in PBS, incubated in a biotinylated secondary donkey anti-mouse antibody (2 h, 1:500 in PBS/0.3% Triton X-100; Jackson Immunoresearch), washed in PBS, then processed for avidin–biotin horseradish peroxidase/3,3´-diaminobenzidine enhanced with nickel (niDAB, Blue-Black). After the primary antibody against the M2 receptor was visualized, sections/explants were washed repeatedly in PBS and then incubated overnight (4°C) in anti-GnRH primary rabbit antibody (SW-1; RRID:AB_2629221). The following day, explants/tissue were washed in PBS, then incubated in Alexa Fluor 555-conjugated secondary donkey anti-rabbit antibody (2 h, 1:1000 in PBS/0.3% Triton X-100; Life Technologies). After washing in PBS and water, explants/sections were coverslipped with Fluoro-Gel (Electron Microscopy Sciences). The specificity of the M2 antibody was evaluated using brain tissue from a M2 knockout mouse (kindly provided by Dr. Jurgen Wess, **Supplemental Figure 1**).

### ChAT afferents to GnRH cells

Free-floating PN10, PN35 and Adult mouse brain sections from male and female animals (ChAT-Cre/Rosa26^tdTomato^/GnRH-GFP) were removed from cryoprotectant (−20°C), rinsed in PBS (1 h), and incubated in blocking solution (2 h, as above). The GnRH-GFP signal was amplified: chicken anti-GFP primary (1:1500 overnight, 4°C, RRID:AB_300798), PBS washes (6x10min), donkey anti-chicken Alexa Fluor 488 (2 h; 1:1000 Jackson Immunoresearch). Sections were mounted on subbed slides, coverslipped with Vectashield Antifade Mounting Media (Vector) and imaged using a spinning disk confocal system CSU10 (Yokogawa), an Eclipse TE200 microscope (Nikon), an EMCCD ImageM digital camera (Hamamatsu), and iVision software (BioVision). Images were processed for viewing using ImageJ (W Rasband, NIH, Bethesda, MD, United States) and Photoshop (Adobe Systems Inc., Salinas, CA) software. Control sections in which one of the primaries was replaced with bovine serum albumin (BSA) showed no detectable signal at that wavelength.

#### Chromogen staining

To facilitate counting the number of GnRH cells potentially modulated by ACh, sections were stained in chromogen to observe ChAT-positive fibers apposed to GnRH neurons. Sections from WT mice were used. Prior to staining, sections were washed in PBS (6x10 min), incubated in 0.3% hydrogen peroxide (15 min), washed in PBS, then blocked, washed (6 x10 min), and incubated (2 nights, 4°C) in anti-ChAT primary goat antibody (1:300, RRID: AB_2079751). Sections were then washed in PBS, incubated in a donkey anti-goat biotinylated secondary (2 h, 1:500 in PBS/0.3% Triton X-100; Jackson Immunoresearch), washed, then processed using standard procedures for avidin–biotin horseradish peroxidase/3,3´-diaminobenzidine enhanced with nickel (niDAB, black). After visualizing the first primary antibody with niDAB, sections were washed (PBS, 6x10 min), incubated in 3% hydrogen peroxide (2x10 min), washed (PBS, 6x10 min), and incubated for two nights in anti-GnRH primary rabbit antibody (4°C, SW-1; RRID:AB_2629221). Sections were washed in PBS, placed in secondary donkey anti-rabbit antibody (2 h, 1:500 in PBS/0.3% Triton X-100; Jackson Immunoresearch) and processed using standard procedures avidin-biotin-horseradish peroxidase/3′3-diaminobenzidine (DAB, brown). Sections were washed in PBS then water, dehydrated in ethanol, placed in xylene, and coverslipped (permount mounting medium, Fisher Scientific). After mounting, the total number of GnRH cells as well as the number of GnRH neurons apposed by ChAT positive fibers was counted in 2/4 serial series (series 1 and 3, N=3 each age and sex). Sections were analyzed at 40X on a brightfield microscope. GnRH cells were counted as positive when either ChAT stained black fibers or large black puncta (representing fibers parallel or perpendicular to the plane) were detected on brown GnRH neurons. The number of GnRH cells with vs without ChAT contacts was counted for each section, summed for the total number of GnRH cells evaluated. GnRH neurons form in a rostro-caudal continuum, centered around the *organum vasculosum of the lamina terminalis* (OVLT). To consider this neuronal spread, the ChAT/GnRH quantification was performed along the rostro-caudal axis using 6 brain regions (spanning over ~2.88 mm). The regions were as follows [span relative to OVLT in µm]: region #1 “rostral MS” [−1080;−360, 9 sections grouped], region #2 “diagonal band/MS” [−360; −120, 3 sections grouped], region #3 “OVLT” [−120; +120, 3 sections grouped], region #4 “crossing of anterior commissure/optic chiasm” [+120;+360, 3 sections grouped], region #5 “suprachiasmatic nucleus” [+360;+600, 3 sections grouped], region #6 “supraoptic nucleus/arcuate nucleus/median eminence” [+600;+1800, 15 sections grouped]. Statistical analysis was then conducted on percent of GnRH cells with ChAT appositions out of the total number of GnRH neurons counted (two-way ANOVA).

#### Phenotype of ACh neurons in MS

ChAT-Cre/Ros26^tdTomato^ adult sections containing the MS were examined for p75NGFR (1:5250, RRID: AB_90760) and substance P (1:32,000, RRID:AB_2922957). Only p75NGFR was co-localized with tdTomato-labeled cells.

#### ChAT/p75NGFR processes on GnRH cells

PN10 and adult brain sections from ChAT-Cre/Rosa-Tomato/GnRH-GFP mice were used. Sections were incubated in rabbit anti-p75NGFR (1:5250, 48 h, 4°C, RRID: AB_90760), washed in PBS, incubated in donkey anti-rabbit Alexa Fluor 647-conjugated (2 h; 1:1000; Jackson Immunoresearch), quickly fixed (10 min, 4% formalin), washed (6x5 min PBS) and subsequently stained for GFP (1:1500 overnight, 4°C, RRID:AB_300798) as described above. Sections were imaged with a spinning disk confocal system CSU10 (Yokogawa), an Eclipse TE200 microscope (Nikon), an EMCCD ImageM digital camera (Hamamatsu), and iVision software (BioVision). Images were processed for viewing using ImageJ (W Rasband, NIH, Bethesda, MD, United States) and Photoshop (Adobe Systems Inc., Salinas, CA) software.

## Results

### ACh alters GnRH neuronal activity

#### ACh evokes a transient calcium influx, *via* nAChR activation and subsequent opening of L-type voltage-gated calcium channels

Exogenous acetylcholine (ACh, 100 µM) was applied to explants and changes in intracellular calcium levels ([Ca^2+^]i) in GnRH neurons were determined using calcium imaging. As *in vivo*, GABAergic and glutamatergic inputs to GnRH neurons are robust in explants (25). As such, excitatory inputs were blocked by treatment with AAB (20 μM BIC, 10 μM CNQX, 10 μM AP5). After a control period (SFM 5 min, followed by AAB 5 min), ACh was added and induced a transient increase in [Ca^2+^]i in ~75% of the GnRH cells returning to baseline levels within 5-6 min (**Figure 1B**). The area under the curve (AUC) was used to define its magnitude. The calcium response in the presence of AAB demonstrates a direct effect of ACh on GnRH neurons. To determine which type of cholinergic receptor was responsible for this calcium response, scopolamine (10 nM, mAChR antagonist, **Figure 1C**) and mecamylamine (10 µM, nAChR antagonist, **Figure 1D**) were used. Blocking mAChRs had no impact on the ACh-induced response, however blocking nAChRs completely prevented it, suggesting that nAChRs are the receptor group mediating cholinergic excitation. In agreement with this, nicotine (100 µM) application mimicked the [Ca^2+^]i time course seen with ACh (**Figure 1E**), though the AUC was greater (**Figure 1F**).

**Figure 2:**
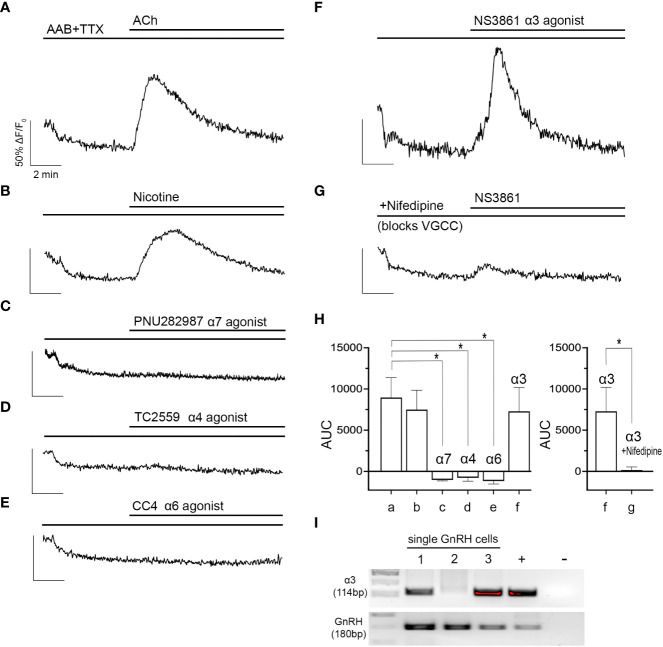
ACh-induced increase in [Ca^2+^]i occurs through activation of the alpha3 nAChR and subsequent opening of voltage gated calcium channels. **(A, B)** ACh (100 µM) and nicotine (100 µM) increases in [Ca^2+^]i in GnRH cells were sustained despite blocking sodium channels with tetrodotoxin (TTX, 0.5 µM) (n=176 and 127). **(C–E)** Agonists (10 µM) to specific nAChR subtypes (alpha7, alpha4, alpha6) did not evoke increase in [Ca^2+^]i (n=148, 118, and 121). **(F)** only NS3861, alpha3 nAChR agonist (10 µM), mimicked ACh- and nicotine- induced increase in [Ca^2+^]i (n=187). **(G)** The alpha3-specific nAChR activation was inhibited by nifedipine (1 µM), indicating ACh-induced increase in [Ca^2+^]i relies upon calcium influx through VGCC (n=153). **(H)** Graphs summarizing AUC values averaged for all explants **(N)**. N=5 for all parameters except nifedipine (N=6). Average number of GnRH cells analyzed/explant ranged from 21-35. a, ACh, b, Nicotine, c, PNU282987, d, TC2559, e, CC4, f, NS3861, g, NS3861+nifedipine. Asterisks represent statistical significance between ACh (control) and other compounds (b-f) (*p*<0.05, one-way ANOVA test with Dunnett’s multiple comparisons; left graph) or between f-g only (*p*<0.05, Student’s *t*-test; right graph). **(I)** transcripts for the alpha3 receptor were found in single cell GnRH neurons. Adult brain and water were used as positive and negative controls respectively. (n = number of cells, N = number of explants).

Nicotinic AChRs consist of an assembly of multiple subunits (8). Because nAChRs are ionotropic receptors (8), spontaneous neuronal activity is not required to assess their activation. Thus, to identify the nAChR(s) involved in the response to ACh, tetrodotoxin (TTX, 0.5 µM) was used in addition to AAB to pause GnRH neuronal activity and flatten baseline [Ca^2+^]i _(_ 25_)._. TTX did not alter the responses to ACh or nicotine (**Figures 2A, B**). Although alpha4 and alpha7-containing nAChRs are the most abundant nAChR forms in the central nervous system (26), neither subunit-specific agonist, TC2559 (10 µM, alpha4 nAChR agonist) nor PNU282987 (10 µM, alpha7 nAChR agonist) nor CC4 (10 µM, alpha6 nAChR agonist) evoked an increase in [Ca^2+^]i (**Figures 2C–E**). In contrast, alpha3 subunit-specific agonist NS3861 (10 µM) induced an increase in [Ca^2+^]i in a similar percent of the GnRH cells and of the same magnitude as those induced by ACh and nicotine (**Figure 2F**).

Although nAChRs are relatively permeable to calcium and sodium, direct calcium influx is not a frequent mode of calcium signaling after nAChR activation. Calcium signaling usually occurs through indirect calcium influx (*via* depolarization and subsequent activation of VGCC) or calcium release (*via* calcium-induced calcium release from intracellular stores) (27). To test for indirect calcium influx, alpha3 subunit-specific agonist, NS3861 (10 µM) was applied with a L-type VGCC blocker, nifedipine (1 µM). With nifedipine, NS3861 failed to evoke an increase in [Ca^2+^]i which indicates the Ca^2+^/Na^+^ influx through alpha3-containing nAChR depolarizes the membrane which, subsequently, activates L-type VGCC and triggers the calcium influx (**Figure 2G**). AUCs are summarized in **Figure 2H**. The presence of the alpha3 was then verified by PCR in 2/3 GnRH cells tested (**Figure 2I**).

### ACh also inhibits GnRH neuronal activity in a subset of cells, *via* M2/M4 mAChRs and subsequent opening of GIRK/Kir3 channels

**Figure 3:**
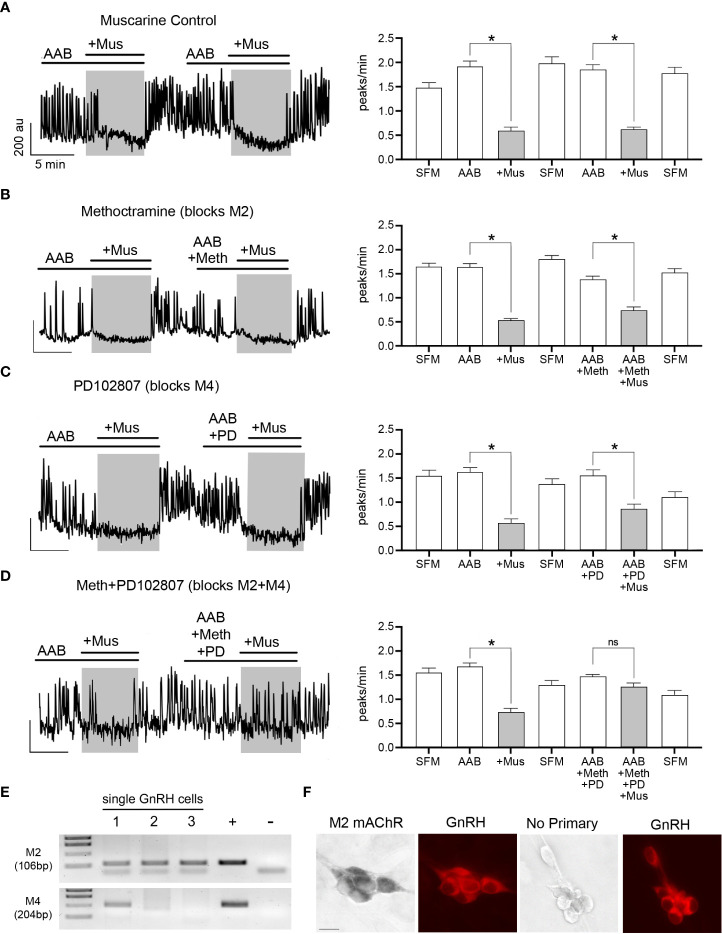
ACh inhibits GnRH neurons through M2/M4 mAChRs. **(A–D)** Left, calcium imaging traces of individual cells, and Right, summary data, showing that muscarine (Mus, 10 µM) significantly reduced the frequency of calcium oscillations in GnRH neurons, calculated as peaks/min (first application). The first muscarine application was used to identify cells inhibited by muscarine (≥0.6 peaks/min decrease), followed by a second muscarine application (grey box) with a coapplied antagonist. Asterisks represent statistical significance between AAB application and subsequent compounds (*p*<0.05, Student’s *t*-test). **(A)** Muscarinic inhibition was repeatable (second application) after a SFM washout (n=50, N=3). Y-axis: au, Arbitrary unit; X-axis: 5 min. Asterisks represent statistical significance between the two consecutive periods of interest with paired Student’s *t*-test, *p*<0.05. **(B, C)** Blocking only M2 or M4 mAChRs with methoctramine (50 nM; n=82, N=4) or PD102807 (400 nM; n=46, N=4), respectively was not sufficient to prevent muscarinic inhibition **(D)** Blocking M2 and M4 mAChRs together prevented the inhibitory effects of muscarine (n=52, N=5). Average number of GnRH cells analyzed/explant ranged from 10-17. **(E)** M2 (CHRM2) and M4 (CHRM4) receptor transcripts found in single cell GnRH neurons (same cells as alpha3 and GnRH, **Figure 2**). Adult brain and water were used as positive and negative controls, respectively. **(F)** Images (left) showing GnRH neurons (red) are immunoreactive for M2 mAChR, (black, niDAB; scale bar, 10 µm). Images (right) show GnRH cells when primary antibodies against M2 mAChR were omitted. * represents statistical significance (p<0.05); ns stands for non significant (p>0.05); (n = number of cells, N = number of explants).

From our initial experiments, when ACh was applied in presence of scopolamine (10 nM, mAChR antagonist), the increase in [Ca^2+^]i was broader (AUC above 50%: ACh=2784 ± 390 (n=6); ACh+scopolamine=5665 ± 430(n=5); Student’s *t*-test, p=0.0008), indicating its termination did not solely rely upon L-type VGCC inactivation but possibly involved concomitant activation of mAChRs. To test this hypothesis, muscarine (10 µM, mAChR agonist) was applied twice upon spontaneously active GnRH neurons, in presence of AAB. Because calcium oscillations are concomitant to bursts of action potentials (21), they are used as a proxy for GnRH neuron firing. The first application identified GnRH cells inhibited by muscarine, and the second tested which mAChR receptor(s) was responsible. Approximately 35% of the GnRH cells showed a significant decrease in the frequency of calcium oscillations during the first application of muscarine, which was repeatable after SFM washout (**Figure 3A**; **Table 2**, row 1, *p*<0.05). Metabotropic mAChRs consist of 5 different subtypes: M1, M3 and M5 subtypes are Gq protein-coupled while M2 and M4 subtypes are Gi protein-coupled (7). To determine which subtype mediates the inhibitory response in GnRH cells, the M2 antagonist methoctramine (50 nM) and M4 antagonist PD102807 (400 nM) were applied (**Figures 3B, C**). Applied alone, neither antagonist prevented inhibition (**Table 2**, row 2 and row 3, respectively, *p*<0.05), however when applied together, the inhibitory effect of muscarine was blocked (**Figure 3D**) (**Table 2**, row 4, p > 0.05). PCR confirmed the presence of M2 and M4 transcripts in GnRH cells (**Figure 3E**) and double label immunocytochemistry detected co-expression of M2 in GnRH cells as well (**Figure 3F**; **Supplemental Figure 1**). Taken together, these data demonstrate activation of both M2 and M4 receptor subtypes mediated ACh-induced inhibition.

**Table 2:** Frequencies of calcium oscillations in GnRH neurons.

Paradigms (N animals)	Period 1: SFM	Period 2: AAB	Period 3: Mus	Period 4: SFM	Period 5: AAB ± Antagonist	Period 6: AAB + Antagonist + Mus (p value)	Period 6: SFM
	Frequencies in peaks/min						
SFM/AAB/+Mus/SFM/AAB/+Mus/SFM (N=3)	1.47 ± 0.12	1.91 ± 0.12	0.59 ± 0.06	1.98 ± 0.14	1.82 ± 0.13	0.59 ± 0.08 (<0.0001*)	1.76 ± 0.14
SFM/AAB/+Mus/SFM/AAB+Meth/+Mus/SFM (N=4)	1.63 ± 0.09	1.64 ± 0.08	0.512 ± 0.06	1.80 ± 0.08	1.37 ± 0.09	0.73 ± 0.08 (<0.0001*)	1.52 ± 0.09
SFM/AAB/+Mus/SFM/AAB+PD/+Mus/SFM (N=4)	1.54 ± 0.12	1.62 ± 0.09	0.57 ± 0.08	1.37 ± 0.11	1.54 ± 0.13	0.85 ± 0.11 (<0.0001*)	1.10 ± 0.11
SFM/AAB/+Mus/SFM/AAB+Meth+PD/+Mus/SFM (N=5)	1.54 ± 0.11	1.67 ± 0.09	0.73 ± 0.08	1.28 ± 0.11	1.41 ± 0.12	1.24 ± 0.10 (0.084 ^ns^)	1.08 ± 0.10
SFM/AAB/+Mus/ SFM+Cs/+AAB/+Mus/SFM (N=4)	1.76 ± 0.11	1.67 ± 0.12	0.60 ± 0.09	2.13 ± .12	1.38 ± 0.10	1.38 ± 0.09 (>0.999 ^ns^)	1.24 ± 0.13
SFM/AAB/+Mus/ SFM/AAB+PMA/+Mus/SFM (N=4)	1.54 ± 0.09	1.16 ± 0.07	0.27 ± 0.05	1.07 ± 0.11	1.93 ± 0.13	1.96 ± 0.12 (0.7817 ^ns^)	2.29 ± 0.14

AAB [BIC 20 µM, CNQX 10 µM, AP5 10 µM], (+)Muscarine Chloride (Mus; 10 µM), Methoctramine Hydrate (Meth; 50 nM), PD102807 (PD; 400 nM), Cesium Chloride (Cs; 5 mM), Phorbol 12-myristate 13-acetate (PMA) (50 nM). Asterisks indicate significant difference comparing period 5 with period 6 (paired Student’s t test; p<0.05; ns, non-significant).

Metabotropic M2/M4 mAChRs are Gi protein-coupled and downstream signaling (7). To reduce cell excitability, they can inhibit adenylate cyclase (7), however this mechanism is ineffective on GnRH neuronal activity (21). The alternative is to activate G-protein coupled inward-rectifying potassium (GIRK/Kir3) channels (28), which is a mechanism commonly used in GnRH neurons (29–32). To determine if M2/M4 mAChRs activate GIRK/Kir3 channels to inhibit GnRH neurons, cells were pretreated for 30 minutes with cesium (Cs, 5mM), a non-specific Kir channel blocker. Cs blocked the inhibitory effect of muscarine (**Table 2**, row 5, p < 0.05), confirming the activation of Kir channels. We then examined which G protein-coupled Kir (GIRK) channels were involved. Tertiapin-Q is the only GIRK/Kir3-specific blocker available, effective on GIRK1/4 subunits (33). Yet, it is ineffective on GnRH neurons which likely express GIRK2/3 subunit combination (29–31). Thus, we triggered protein kinase C phosphorylation, known to inhibit GIRK2-mediated currents (34), by applying a protein kinase C activator, phorbol 12-myristate 13-acetate (PMA, 50 nM). PMA successfully prevented muscarine-induced inhibition (**Table 2**, row 6, p < 0.05), indicating that mAChRs in GnRH neurons couple to GIRK channels to achieve cholinergic inhibition.

### ChAT fibers contact GnRH neurons in an age and sex dependent manner

**Figure 4:**
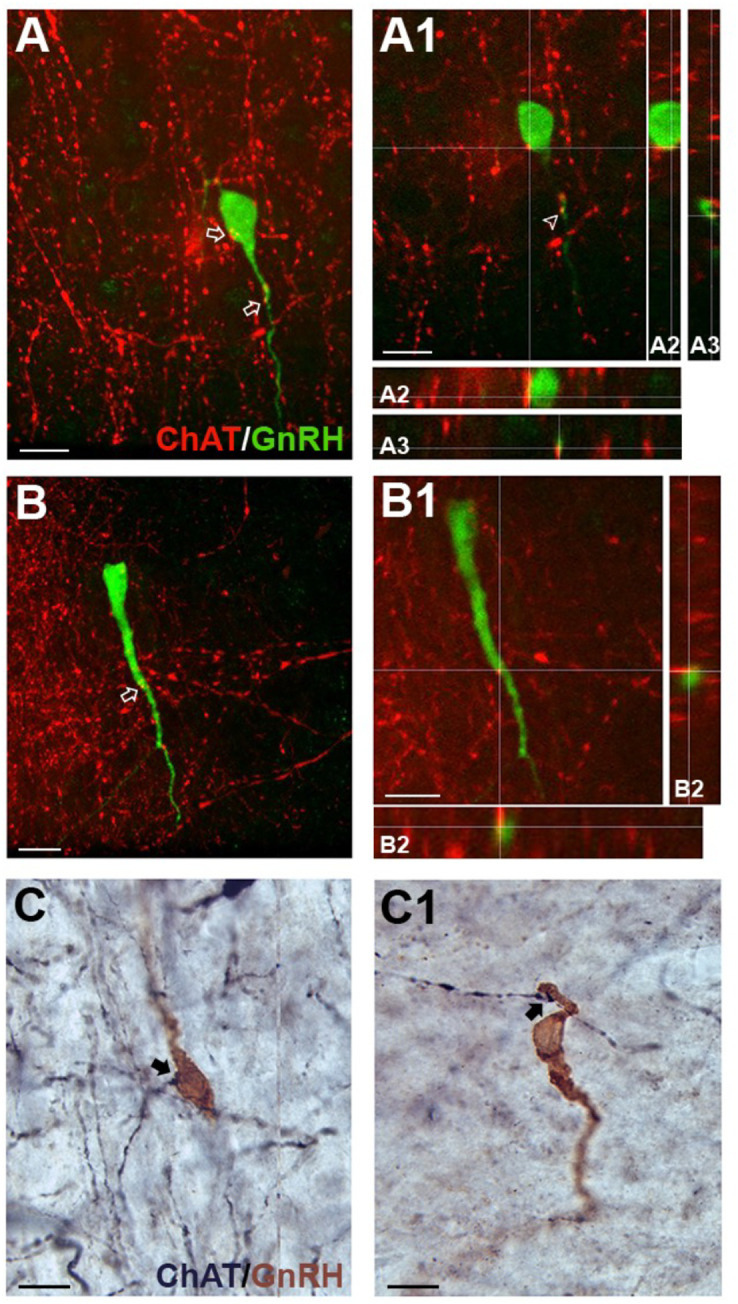
ChAT fibers in the medial septum (MS) contact GnRH neurons. **(A, B)**, tdTomato-labeled ChAT fibers (red) contacting GnRH cell soma (green, **A**) and proximal process **(A, B)**. Arrows point to contacts shown in A1 and B1 respectively. A1, B1, Single z-plane of ChAT fibers contacting GnRH cell soma (shown in A2, y- and x- axis) and proximal process (shown in A3 and B2, y- and x-axis). (C-C1) Two brightfield examples of immunopositive ChAT fibers (black, niDAB) apposing GnRH (brown, DAB) cell soma **(C)** and proximal process (C1) in the MS. Scale bar = 15 μm in **(A-C)**.

ChAT fibers contacting GnRH neurons have been found in adult rat tissue (6). To understand the relevance of cholinergic signaling to GnRH neurons in mouse, the anatomical system underlying cholinergic innervation of GnRH neurons needed to be identified. GnRH/ChAT double immunofluorescent labeling was conducted on brain sections from postnatal (PN10), pubertal (PN35), and adult male and female mice. Using confocal microscopy on mouse sections, ChAT fibers were observed contacting GnRH neurons at all three ages and in both sexes (**Figures 4A, B**). Since GnRH cells are widely distributed throughout the forebrain, we used double immunochemical labeling (**Figure 4C**) to quantify the number and location of GnRH neurons contacted by ChAT fibers. For this analysis, we used the term apposed, rather than contact. Overall, the total number of GnRH cells counted was not significantly different by sex or age (sex: *p*=0.42; age: *p*=0.27). However, since the number of GnRH cells varies across the forebrain (**Supplemental Table 1**), the percent of GnRH cells apposed by ChAT fibers was broken down per brain regions to assess the relationship between GnRH neurons and ChAT fibers (rostral to caudal): region #1 “rostral MS”, region #2 “diagonal band/MS”, region #3 “organum vasculosum lamina terminalis”, region #4 “crossing of anterior commissure/optic chiasm”, region #5 “suprachiasmatic nucleus”, region #6 “supraoptic nucleus/arcuate/median eminence” (**Table 3**). Two-way ANOVA indicated significant differences in GnRH/ChAT relationship between brain regions (p<0.0001) as well as age (*p*=0.0087).

**Table 3:** Chromogen Analysis: Percent ChAT fibers apposed to GnRH Cells.

Brain regions	Females			Males		
	PN 10	PN 35	Adult	PN 10	PN 35	Adult
**Region1 - rostral MS**	40.54 ± 12.6	47.18 ± 11.32	52.95 ± 4.47	15.39 ± 3.99	39.77 ± 6.53	24.19 ± 12.14
**Region 2 - diagonal band/MS**	56.04 ± 5.94	45.57 ± 5.73	40.71 ± 4.81	32.24 ± 2.64	50.88 ± 7.63	43.63 ± 1.45
**Region 3 - OVLT**	30.50 ± 4.63	36.59 ± 13.63	13.71 ± 1.70	28.93 ± 10.56	39.80 ± 6.21	24.04 ± 4.35
**Region 4 - crossing of anterior commissure/optic chiasm**	9.74 ± 7.23	21.47 ± 9.35	5.72 ± 3.04	14.00 ± 11.08	12.09 ± 1.46	20.95 ± 3.9
**Region 5 - suprachiasmatic nucleus**	8.21 ± 5.08	11.72 ± 2.76	3.8 ± 2.22	4.90 ± 3.54	16.59 ± 5.53	8.33 ± 4.81
**Region 6 - supraoptic nucleus/arcuate nucleus/median eminence**	6.67 ± 6.67	11.06 ± 1.97	5.68 ± 3.65	1.04 ± 1.04	7.50 ± 1.15	12.14 ± 1.57

Analysis of regions indicated that the caudal-most regions #4, 5 and 6 (anterior commissure crossing to caudal) were similar and did not show sex or age differences. On average, only 9% of GnRH cells in these caudal regions were apposed by ChAT fibers. In contrast, rostral-most regions #1-3 showed a higher percent of GnRH neurons with such appositions (**Table 3**). Two-way ANOVA revealed regional differences (*p*=0.0045), and a brain region/sex interaction (*p*=0.0321). Analysis of each area, independent of age, showed that in region #1, females had more GnRH neurons apposed by ChAT fibers than males (females 47%, males 26%; *p*=0.0224). In regions #2 and #3, no sex differences were detected. The percent of GnRH cells apposed by ChAT fibers was ~45% (females 47%, males 42%; *p*=0.24) and ~30% (females 27%, males 31%; *p*=0.55), respectively. Age-related changes were then assessed in males and females in these same regions. In females, no change was detected with age (*p*=0.5183). This is in contrast to males (*p*=0.0162). The percent of GnRH neurons apposed by ChAT fibers transiently increased at PN35 in males (Regions #1-3: 40%, 51%, 40%), compared to PN10 (Regions #1-3: 15%, 32%, 29%) and adult (Regions #1-3: 24%, 44%, 24%).

### ChAT fibers originate from p75NGFR septal cholinergic interneurons

**Figure 5:**
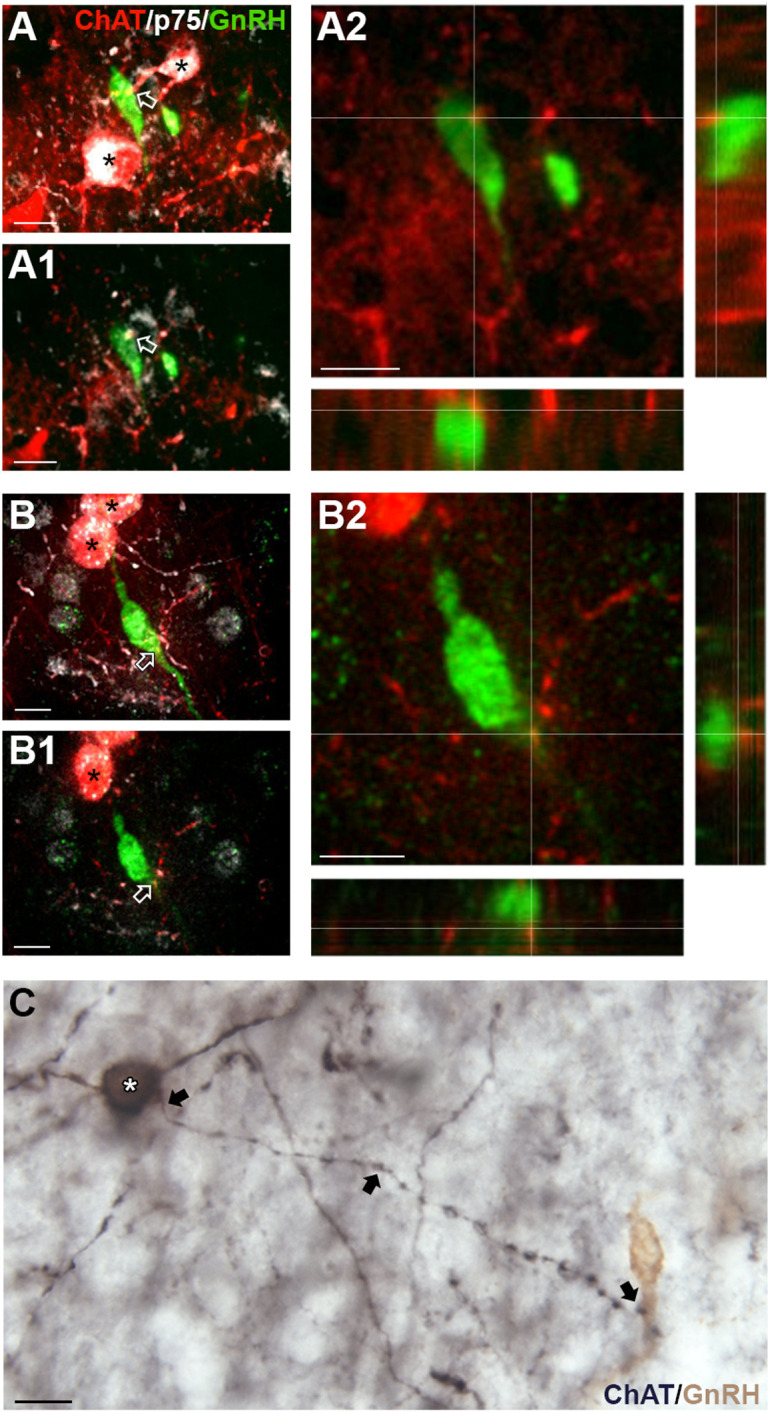
Medial septum (MS) ChAT fibers contacting GnRH neurons express p75NGFR. **(A, B)** Flattened z-stack of tdTomato-labeled ChAT (red), p75NGFR (white) and GnRH (green) in the MS. tdTomato-labeled ChAT cells are p75NGFR positive (asterisks). (A1, B1), Single plane image from z-stack shown in **(A, B)** respectively. Arrows in **(A)** A1 and **(B)** B1 indicate contacts shown in A2 and B2. (A2, B2), Two channel images, showing higher magnification of single plane shown in A1 and B1 respectively. Images highlight tdTomato-labeled ChAT fibers (red) contacting GnRH (green) cell soma **(A)** and proximal process **(B)**. **(C)** Brightfield image showing a p75NGFR positive cell in the MS (white asterisk, black, niDAB) extending a fiber (arrows) that apposes a GnRH neuron (brown, DAB, Composite of two photographs to follow fiber from p75NGFR cells soma to GnRH cell. Scale bar = 15 μm in **(A–C)**.

**Figure 6:**
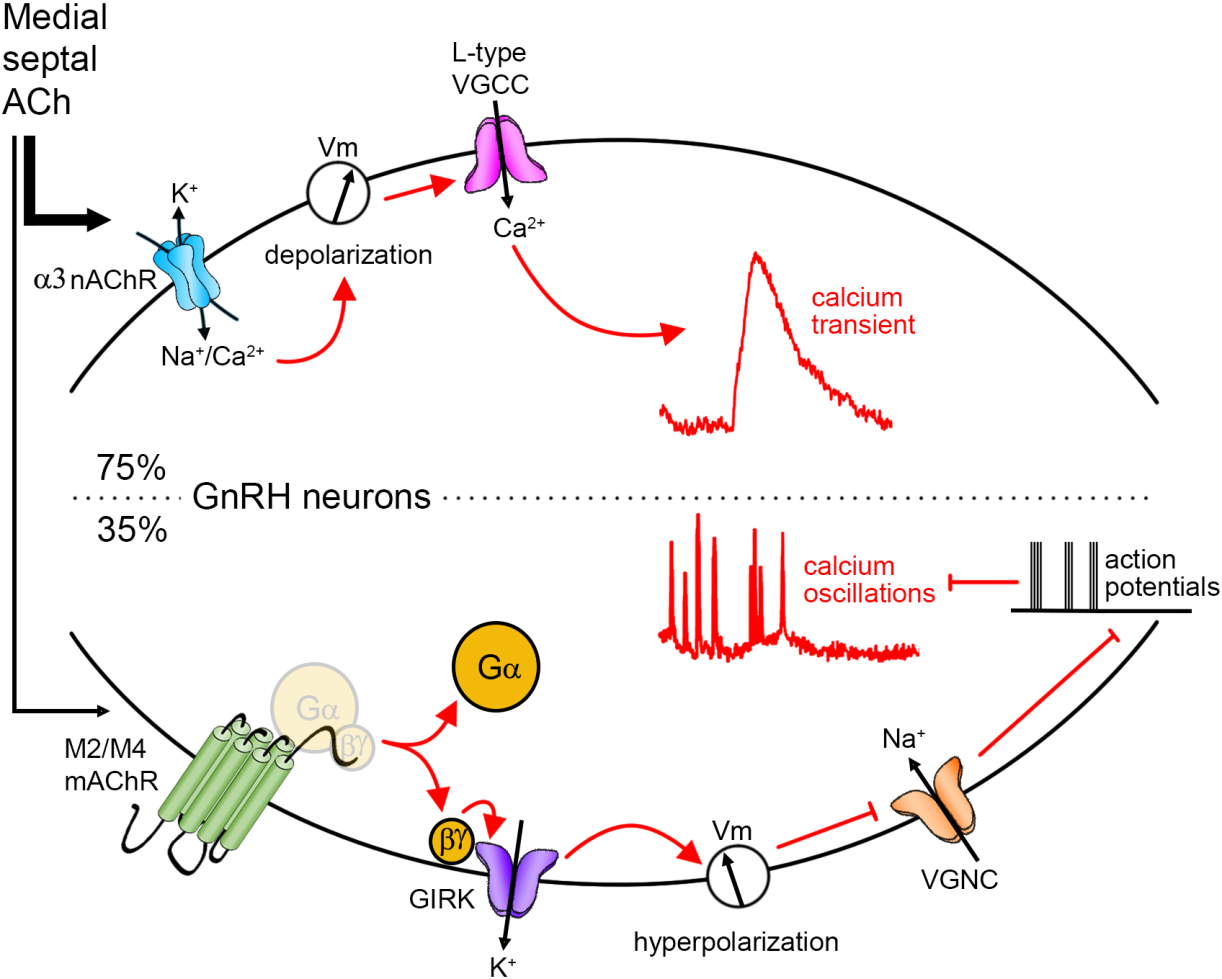
Cholinergic regulation of GnRH neurons. Anatomically, cholinergic afferents to GnRH neurons were identified in the medial septum. Using explant, physiological experiments revealed that upon application of ACh, 75% of GnRH neurons display a calcium transient. The signaling pathway is as follows: the activation of alpha3 nicotinic AChR (nAChR) drives a depolarization in the membrane potential (Vm) that opens voltage-gated calcium channels (VGCC), and subsequently, triggers calcium influx. Some GnRH neurons (35%) display a decrease in GnRH neuronal activity. The signaling pathway is as follows: the activation of M2/M4 muscarinic AChR (mAChR) triggers the dissociation of G protein and the betagamma subunit activates G protein-gated inwardly rectifying potassium (GIRK) channels. The potassium conductance triggers a hyperpolarization which impedes the activation of voltage-gated sodium channels (VGNC). Action potentials and concomitant calcium oscillations are prevented.

Cholinergic fibers predominantly originate from eight brain regions known as Ch1-Ch8 (35). Retrograde tracing shows possible locations with cholinergic fibers projecting to the MS are Ch6, the laterodorsal tegmental nucleus (LDT) in the brainstem (“Allen Brain Atlas Mouse Connectivity - experiment 67151656: AAV-EGFP in laterodorsal tegmental nucleus from Chat-IRES-Cre-neo,” n.d.), and/or Ch1, the MS itself (“Allen Brain Atlas Mouse Connectivity - experiment 156819600: AAV-EGFP in MS from Chat-IRES-Cre-neo,” n.d.). To determine if the LDT was a source, immunofluorescent labeling for substance P, a neuropeptide present in the MS (36) and expressed by LDT cholinergic neurons (37), was conducted on ChAT-Cre/Rosa26^tdTomato^ tissue. Staining showed that ChAT cell bodies/fibers were not colabeling with substance P (data not shown), suggesting that innervation of GnRH neurons is not from LDT substance P/cholinergic neurons. To determine if the MS was the source of cholinergic innervation, immunofluorescent labeling for p75NGFR, known to be expressed by septal cholinergic neurons (38–40), was performed on ChAT-Cre/Rosa26^tdTomato^ tissue. Staining showed that p75NGFR colabeled most of the cholinergic interneurons throughout the MS (**Figure 5**), and that ChAT fibers contacting GnRH neurons were colabeled with p75NGFR (**Figures 5A, B**) and p75NGFR afferents from MS neurons apposed MS GnRH neurons (**Figure 5C**). These data indicate that MS interneurons are one source of direct cholinergic modulation of GnRH neurons. A schematic summarizing the findings described in this paper are shown in **Figure 6**.

## Discussion

This study establishes two mechanisms by which ACh can regulate GnRH neuron activity. One, linked to alpha3-containing nAChRs, which triggers membrane depolarization and subsequent calcium influx *via* opening of L-type VGCC. The other, linked to M2/M4 mAChRs, which activates GIRK channels to inhibit spontaneous calcium oscillations. In the MS, a sex difference was found in the number of ChAT fibers apposed to GnRH cell soma, being greater in females than males at prepubertal and adult stages. Immunofluorescence indicated that the ChAT fibers contacting GnRH cell bodies co-expressed p75NGFR, a signature of cholinergic cells in the MS. While this cholinergic group projects to the hippocampus and plays a critical role in learning and memory performance (41), our data indicate that it also provides a direct cholinergic input to a subpopulation of GnRH neurons and therefore could modulate GnRH neuronal activity and subsequently reproductive function.

### Physiological action on GnRH neuronal activity

Both the increase in [Ca^2+^]i with ACh/nicotine and the muscarine-induced inhibition of spontaneous calcium oscillations we observed in primary GnRH cells are in agreement with *in vitro* data from: 1) hypothalamic fragments in which ACh-induced GnRH secretion was prevented by a nAChR antagonist (14), and 2) the GT1-7 ceIl line in which ACh induced an increase in [Ca^2+^]i (42), nicotine induced GnRH secretion (15), and muscarine inhibited GnRH secretion (16). However, the nAChR subunit and the mAChR we identified here in primary GnRH cells for stimulation and inhibition, respectively, differ from those identified in GT1-7 cells. The alpha3 nAChR subunit was responsible for the ACh/nicotine response in primary GnRH cells while in the GT1-7, the alpha7 nAChR subunit was linked to nicotine’s effects on GnRH secretion (increased or decreased) (15, 17). The muscarine-induced inhibition of spontaneous calcium oscillations we observed was triggered by both the M2/M4 mAChRs interchangeably, whereas only M2 mAChR was associated with muscarine inhibition in GT1-7 cells (16).

Certainly the status of immortalized GT1-7 cells might alter gene expression (43). RNA sequencing data from GnRH neurons from adult mice show high levels of the *Chrna3* (gene for alpha3 nAChR), but low levels of expression for *Chrna7* (gene for alpha7 nAChR), and high levels of expression for *Chrm2*/*Chrm4* (genes for M2 and M4 mAChRs, respectively) (44), which support our observation in native GnRH neurons. Yet, this database also indicates high levels of expression for *Chrna4* (gene for alpha4 nAChR) and *Chrm1* (gene for M1 mAChR), which we did not functionally detect. This might be due to the subcellular region imaged here, i.e. the cell body. For example, in both retinal ganglion cells and serotoninergic neurons in the dorsal raphe nucleus, alpha4 nAChRs are localized on dendrites (45, 46). In the cerebral cortex and hippocampus, M1 mAChRs are also rarely observed on cell bodies, being preferentially located on dendrites and spines of pyramidal cells (47). Supporting a possible role for distal regulation, muscarinic stimulation mostly evokes GnRH release from median eminence fragments (48). Thus, AChRs other than those identified here might regulate GnRH neurons in different subcellular compartments.

The downstream signaling pathways we describe after the activation of alpha3-containing nAChR and M2/M4 mAChRs are canonical. Nicotinic AChRs, including those containing the alpha3 subunit, commonly activate of VGCC (27). Muscarinic AChRs, including the M2 and M4 subtypes, commonly activate GIRK channels (28). In our hands, M2 and M4 mAChRs were interchangeably effective at inhibiting GnRH neurons. This is also seen in cholinergic neurons in the brainstem in which the muscarinic inhibition occurs *via* either muscarinic-subtype activating GIRK channels (49). An additional inhibitory signaling pathway downstream of M4 mAChR exists in striatal neurons *via* VGCC modulation (50). However, this cannot account for the inhibition of calcium oscillations in GnRH neurons since VGCC, including L-type, do not participate in calcium oscillations (51). Yet, this could be the mechanism, in addition to the activation of GIRK channels, by which mAChRs control the duration of calcium influx evoked by nAChR/VGCC.

Alpha3 nAChRs are found in specific brain areas, including the hypothalamus (52, 53) (and references within). Genetic deletion of alpha3 nAChR is lethal (54). Histological studies of alpha3 nAChR KO mice revealed no significant abnormalities in brain and lethality was determined to be due to abnormal function of the autonomic nervous system (54). Overall, its role in specific physiological brain functions remain unknown. Our data indicate that activation of alpha3 nAChRs on GnRH neurons exhibit a slow desensitization. This could increase GnRH neuronal excitability, as reported in vasoactive intestinal peptide-expressing neurons (55). However, the massive calcium influx with ACh/nicotine/alpha3 agonists could trigger calcium-activated potassium channels which would subsequently decrease excitability. Our data support the latter, which is in agreement with the role of these channels in patterning action potentials in GnRH neurons (56, 57).

Physiologically, the role of M2/M4 mAChRs on the GnRH cell soma might act as a brake on GnRH neuron excitability, being near the axon initial segment (58). However, how such a brake might influence female cyclicity is unclear due to the lack of cell specificity targeted by central drug delivery used in the *in vivo* studies (9–12) and few studies have reported changes in reproductive behavior in males. However, ChAT mRNA levels fluctuate during the estrous cycle, being the highest during diestrus, in response to high estrogen levels in proestrus (59). RNA sequencing data from GnRH neurons suggest estrogen-dependent regulation of expression levels of *Chrm2*/*Chrm4* (44) (encoding M2/M4), which is not seen with genes coding for any nAChRs. Together, this could indicate that the maximal inhibition of GnRH neuron excitability by ACh would occur after the preovulatory GnRH/LH surge.

### ACh afferents to GnRH neurons

In agreement with previous data in rats (6), we found ChAT positive fibers contacting GnRH cell bodies and extended the analysis to prepubertal, pubertal and adult male and female mice. The nature of these cell-to-cell appositions was not determined as cholinergic activation can be synaptic and extrasynaptic, *via* volume transmission (60). A greater number of ChAT fibers apposed to GnRH cell soma was found in the MS of females compared to males, at PN10 and in adults. However, at puberty, males exhibited a transient increase in ChAT appositions to GnRH neurons in this area, equivalent to that detected in females across all three stages.

There are two primary cholinergic centers in the brain (61, 62): the brainstem and the basal forebrain. The brainstem cholinergic system consists of the pedunculopontine tegmental nucleus (PPT) and the laterodorsal tegmental nucleus (LDT). No connections from the PPT to the septal area have been reported. The LDT cholinergic cells, which send afferents to the lateral septum (63), are involved in vocalization associated with emotional arousal, such as following mating (64). Since many of these LDT cholinergic cells express substance P (65), ChAT fibers around GnRH neurons in the MS were stained for substance P to test for their presence. No substance P/ChAT fibers were detected, confirming cholinergic LTD cells are not inputs to GnRH neurons. In contrast, immunolabeling indicated that these fibers co-expressed p75NGFR, a signature of cholinergic cells in the MS (66). This cholinergic group is well known to project to the hippocampus and play a critical role in learning and memory performance (67–69). However, our data indicate a distinct subpopulation of GnRH neurons receive direct cholinergic signaling from MS ChAT/p75NGFR neurons.

Few studies of ChAT-expressing cells in general, and certainly within the MS, have examined pre-, pubertal and adult stages in male and female mice. However, it is clear in adult rats and mice that ChAT/p75NGFR immunoreactive neurons in the MS also express estrogen receptors in both females (70, 71) and males (71). Furthermore, while androgen receptors are not detected in ChAT-expressing cells in the MS of adult male rats (72), some cells express P450 aromatase mRNA (73). Counts of cholinergic cells in the MS revealed no difference between males and females in adult rats (71) or mice (74). However, it is known that the levels of gonadal steroids affect the number of ChAT neurons in the MS (72), the enzymatic activity of ChAT (4) and alter spine density and afferent connections to GnRH neurons (75–77). In the present study, the percent of GnRH cells apposed by ChAT-expressing fibers in females remained constant across prepubertal, pubertal and adult time points. In contrast, the percent of GnRH cells apposed by ChAT fibers in males showed a transient increase in pubertal mice which could be due to the pubertal elevation of circulating gonadal hormones (78). Although the percent of GnRH cells apposed by ChAT fibers decreased back to prepubertal levels in adult males as circulating gonadal hormones remain elevated (78), the pubertal increase in ChAT fibers apposed to GnRH cells in males demonstrate that gonadal steroid feedback might modulate the cholinergic output of MS interneurons. While data support sex differences in cognitive function in Alzheimer’s patients and animal models due cholinergic projections to the hippocampus (79, 80), further experiments in which gonadal steroid levels are controlled are needed, to understand how cholinergic regulation is entwined with reproductive function.

## Data availability statement

The original contributions presented in the study are included in the article/**Supplementary Material**. Further inquiries can be directed to the corresponding author.

## Ethics statement

The animal study was reviewed and approved by All procedures were approved by National Institute of Neurological Disorders and Stroke, Animal Care and Use Committee and performed in accordance with National Institutes of Health guidelines.

## Author contributions

SC and SW conceptualized and designed experiments. DS and JF performed experiments. SC, DS, JF and SW analyzed data. SC and SW finalized the manuscript. All authors contributed to the article and approved the submitted version.

## Supplementary material

Supplementary Figure 1 Validation of antibody against M2 mAChR. Double labeling for M2 (black, niDAB) and GnRH (Alexa Fluor 488-conjugated secondary). **(A)** Staining for M2 mACHR immunoreactive cells was detected in wild type (WT) tissue, but no staining was detected in tissue from M2 mAChR knockout (M2KO) or WT mouse tissue in which the primary antibody (M2) was replaced with BSA. **(B)** In contrast, GnRH cells were detected in all tissues after M2 staining. Scale bar in first panel = 100 µm in all low magnifications and 10 µm in insets.
