# Supplementary figures and images for: Acetylcholine regulation of GnRH neuronal activity: A circuit in the medial septum
Source: Front Endocrinol (Lausanne). 2023 Mar 6;14:1147554. doi: 10.3389/fendo.2023.1147554 (PMC10025473; doi:10.3389/fendo.2023.1147554)

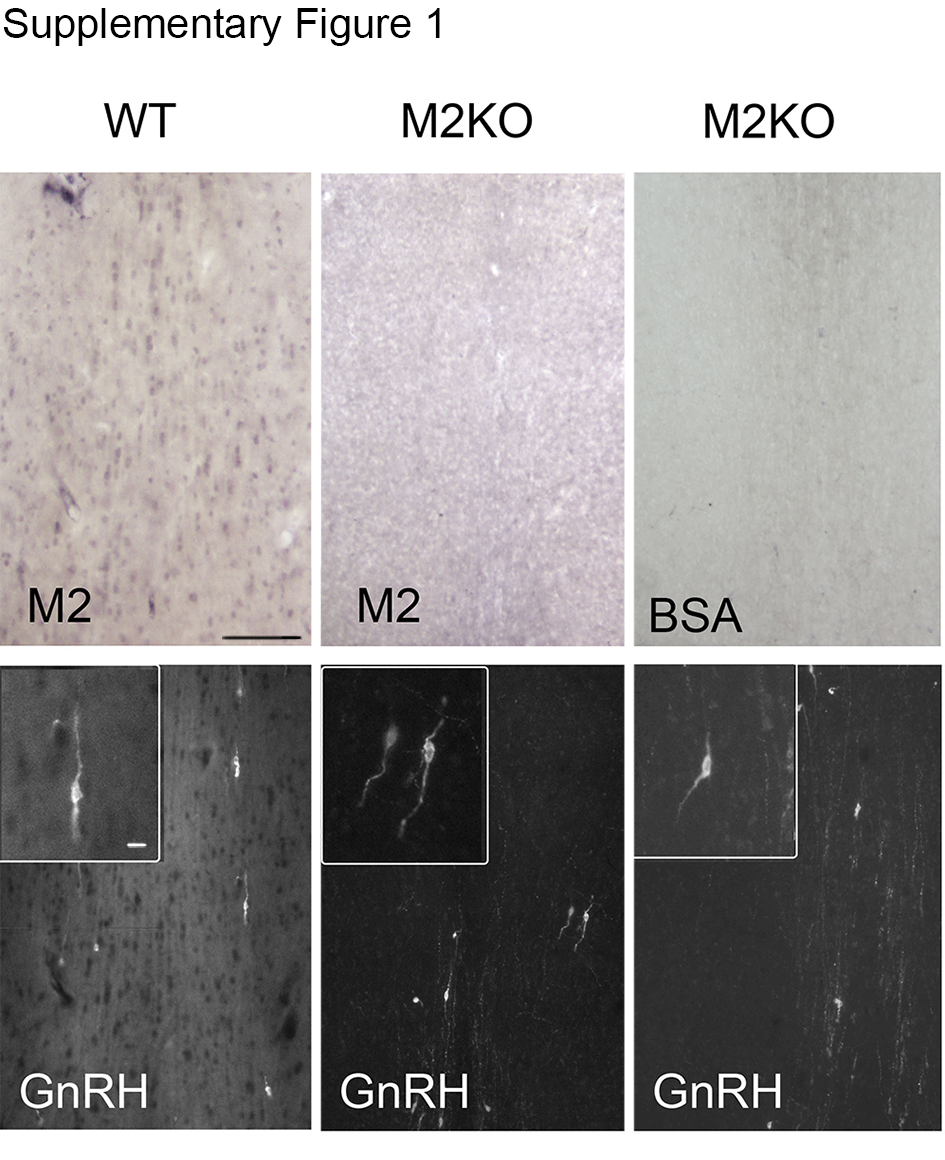

Supplement: Supplementary Figure 1 — Validation of antibody against M2 mAChR. Double labeling for M2 (black, niDAB) and GnRH (Alexa Fluor 488-conjugated secondary). (A) Staining for M2 mACHR immunoreactive cells was detected in wild type (WT) tissue, but no staining was detected in tissue from M2 mAChR knockout (M2KO) or WT mouse tissue in which the primary antibody (M2) was replaced with BSA. (B) In contrast, GnRH cells were detected in all tissues after M2 staining. Scale bar in first panel = 100 µm in all low magnifications and 10 µm in insets. [file Image_1.tif]
