# Supplementary material for: Acetylcholine regulation of GnRH neuronal activity: A circuit in the medial septum
Source: Front Endocrinol (Lausanne). 2023 Mar 6;14:1147554. doi: 10.3389/fendo.2023.1147554 (PMC10025473; doi:10.3389/fendo.2023.1147554)
Supplement: Supplementary file 2 [file Table_1.docx]

**Supplementary Table 1. Total number of GnRH cell counted/area in 2/4 series**

| **Brain regions** | **Females** | | | **Males** | | |
| --- | --- | --- | --- | --- | --- | --- |
|  | **PN 10** | **PN 35** | **PN 10** | **PN 35** | **PN 10** | **PN 35** |
| **Region1 - rostral MS** | 60.33±22.58 | 48.33±4.18 | 36±7.51 | 49.67±17.83 | 62.67±17.98 | 36.67±8.37 |
| **Region 2 - diagonal band/MS** | 42.67±8.25 | 39.67±2.60 | 53.67±11.85 | 49±11.93 | 39.67±2.60 | 42.33±11.17 |
| **Region 3 - OVLT** | 71.33±11.8 | 48.33±6.69 | 72.67±9.56 | 88±23.8 | 52±5.69 | 94.67±24.4 |
| **Region 4 - crossing of anterior commissure/optic chiasm** | 71.33±20.93 | 45.67±10.27 | 50.67±16.05 | 76±17.44 | 83±14.22 | 33.67±6.17 |
| **Region 5 - suprachiasmatic nucleus** | 32.67±3.71 | 28.33±3.48 | 16±5.69 | 31.67±2.33 | 37.33±7.88 | 17.33±6.67 |
| **Region 6 - supraoptic nucleus/arcuate nucleus/median eminence** | 47.67±16.76 | 36±9.17 | 36.67±8.17 | 35.33±9.6 | 57±2.08 | 38.67±12.35 |
